# From Rib Hump to Baby Hump—Common Questions of Patients Suffering from and Undergoing Treatment for Scoliosis—A Comprehensive Literature Review

**DOI:** 10.3390/jcm13133814

**Published:** 2024-06-28

**Authors:** Pawel Grabala, Piotr Kowalski, Michal Grabala

**Affiliations:** 1 Department of Pediatric Orthopedic Surgery and Traumatology, University Children’s Hospital, Medical University of Bialystok, Waszyngtona 17, 15-274 Bialystok, Poland; 2 Department of Neurosurgery, Regional Specialized Hospital, ul. Dekerta 1, 66-400 Gorzow Wielkopolski, Poland; pkowal72@gmail.com; 3 2nd Clinical Department of General and Gastroenterogical Surgery, Medical University of Bialystok, ul. M. Skłodowskiej-Curie 24a, 15-276 Bialystok, Poland; michal@grabala.pl

**Keywords:** idiopathic scoliosis, AIS, adolescent idiopathic scoliosis, pregnancy, motherhood, scoliosis and pregnancy, scoliosis childbearing, scoliosis and delivery, pregnancy back pain

## Abstract

**Background**: Scoliosis, the most prevalent spinal deformity in children, is often associated with adolescent idiopathic scoliosis (AIS). Curves surpassing 50 degrees can deteriorate, especially thoracic curves, leading to pulmonary limitations. Surgical intervention is usually advised for curvatures exceeding 50 degrees to halt further progression. Severe AIS is notably more frequent in females, yet knowledge regarding subsequent pregnancies and associated risks is scarce. Female patients often inquire about conception, pregnancy, labor, and future back pain (BP) concerns. Reports on the long-term outcomes after pediatric AIS treatment and pregnancy consequences remain limited. Uncertainty looms over the likelihood of increased back pain (BP), cesarean sections (CSs), or other pregnancy-related issues following surgical AIS management. In this investigation, an attempt was made to scrutinize the existing research on individuals afflicted with scoliosis who received either conservative or surgical treatment, with the aim of delineating crucial and pragmatic findings that can serve as a compass for spine surgeons when counseling young patients with adolescent idiopathic scoliosis regarding the ailment, its repercussions, the available treatment modalities, and the associated outcomes. A comprehensive analysis pinpointed the optimal data at hand. Consequently, the primary objective of this investigation was to assess the patient-reported and clinical consequences in scoliosis patients who have undergone segmental posterior fusion and subsequently conceived. **Conclusions:** While the majority of individuals with AIS are capable of conceiving and bearing children, they may encounter greater challenges in fertility than healthy women unaffected by AIS. Pregnant women with a prior history of spinal fusion for AIS can undergo pregnancy and childbirth with no significant escalation in perinatal complications. Women who have undergone surgical procedures for AIS have been observed to exhibit a prevalence of back pain comparable with that of healthy pregnant women; however, a higher incidence of low back pain is evident when spinal fusion is extended to the L3 or L4 vertebra. Although back pain is a common occurrence in pregnant women with AIS, the spinal alterations induced by pregnancy are typically minor and transient. In comparison with healthy women, individuals with a history of spinal fusion necessitate cesarean sections more frequently. The degree of correction loss during pregnancy is lower in previous reports involving pedicle screw instrumentation than in previous reports involving Harrington or hybrid segmental instrumentation.

## 1. Introduction

Scoliosis, the most prevalent spinal deformity in children, is often associated with adolescent idiopathic scoliosis (AIS), marked by a lateral spine curvature of 10 degrees alongside vertebral rotation due to the definition of Scoliosis Research Society. Diagnosis is primarily exclusionary, confirmed after ruling out other scoliosis causes. A 10-degree curvature affects 2% to 3% of children under 16, with 0.3% to 0.5% progressing to a 20-degree curvature, prompting treatment. Moderate curves exceeding 30 degrees appear in about 1–3% of 1000 adolescents upon skeletal maturity [1,2,3]. Curves surpassing 50 degrees can deteriorate, especially thoracic curves, leading to pulmonary limitations [4,5,6,7]. Surgical intervention is usually advised for curvatures exceeding 50 degrees to halt further progression [8,9]. Severe AIS is notably more frequent in females, yet knowledge regarding subsequent pregnancies and associated risks is scarce [1,3,10,11]. Patients often inquire about conception, pregnancy, labor, and future back pain (BP) concerns. The lack of comprehensive data hinders preoperative decision-making, which impacts patient contentment and anticipations [11,12,13]. Reports on the long-term outcomes, health-related quality of life (HRQoL) after AIS treatment and pregnancy consequences remain limited. Uncertainty looms over the likelihood of increased BP, cesarean sections (CSs), or other pregnancy-related issues following surgical AIS management. Data scarcity persists on BP or CS occurrences following segmental spinal instrumentation for AIS.

In this investigation, an attempt was made to scrutinize the existing research on individuals afflicted with scoliosis who received either conservative or surgical treatment, with the aim of delineating crucial and pragmatic findings that can serve as a compass for spine surgeons when counseling young patients with adolescent idiopathic scoliosis regarding the ailment, its repercussions, the available treatment modalities, and the associated outcomes. A methodical exploration of pertinent literature was carried out across various electronic databases, such as EMBASE, PubMed, ScienceDirect, Web of Science, The Cochrane Library, Google Scholar, and Ovid MEDLINE. The search strategy included diverse combinations of keywords like “scoliosis and pregnancy” and incorporated Mesh and non-Mesh terms such as “scoliosis”, “spinal curvature”, “cobb angle”, “spinal deformity”, “adolescent idiopathic scoliosis”, “idiopathic scoliosis”, “adolescent scoliosis”, “pregnant”, “pregnancy”, “childbearing”, “childbirth”, “pregnancy complications”, “birth”, “antenatal”, “perinatal”, “postpartum”, “caesarean”, “pregnancy disorders”, “labor”, “delivery”, and “obstetric complications”. The most recent search was executed on 31 December 2023, and further pertinent studies were recognized through manual scrutiny of the references in the obtained publications. The criteria for inclusion encompassed original research studies, case reports, systematic reviews, and meta-analyses that were published in the English language for abstracts. To prevent redundancy, if a particular study population was presented in multiple publications, the most recent one was considered for inclusion. Exclusions were applied to stand-alone abstracts, articles containing duplicated data, and those lacking original data. Publications that did not meet specific research criteria or lacked transparent methodologies were also excluded. The aim of the study was to analyze patients operated on using techniques based on Cotrel–Dubousset instrumentation, but due to the small amount of available data, we also had to rely on publications based on the results of treatment using the Harrington instrumentation. Data extraction was performed by two independent authors who collected information on the country, primary author, publication year, study population particulars, intervention, study design, and duration of follow-up. Any discrepancies were resolved through mutual consensus discussions.

Owing to the restricted availability of studies concerning the prenatal and postnatal periods, the focus was directed towards studies elucidating spinal alterations attributed to pregnancy, childbirth, and the unique difficulties encountered by these individuals. A comprehensive analysis has pinpointed the optimal data at hand. Consequently, the primary objective of this investigation was to assess the patient-reported and clinical consequences in scoliosis patients who have undergone segmental posterior fusion and subsequently conceived.

## 2. Ability to Get Pregnant

In the literature of retrospective analyses, the percentage of nullipara females among patients with idiopathic scoliosis who underwent either conservative or surgical management varies. A study by Cochran demonstrated that 56% of scoliotic women in Sweden had never given birth, a proportion comparable with their healthy counterparts [14]. Various other retrospective studies have indicated a wide range of nulliparous women in AIS cases, spanning from 13% to 70% [11,15,16,17]. Danielsson et al. conducted research involving surgically and corset-treated women, revealing no disparities in childbirth numbers when juxtaposed with a control group without AIS [18]. Conversely, Akazawa et al. identified a lower offspring count in patients with idiopathic scoliosis versus healthy females [19]. Other investigations reported a childbirth rate of 1.4 children per mother among these patients, without a control group reference [11,15]. The comparative analysis of surgically managed participants [19,20,21] displayed no significant variations in the nulliparous women count or pregnancy rate concerning a healthy control group or the national norm [16,20,22]. When contrasting surgically managed subjects with conservatively managed ones, no notable differences were observed in terms of childless individuals or those with children, with an average of 1.8 children in surgically treated cases, 1.9 in brace-treated cases, and 2.0 in the healthy cohort [15,16,18,22,23]. The average maternal age at the time of first delivery ranged from 26 to 33 years, remaining consistent between AIS mothers and their healthy counterparts [18,24,25]. Notably, one study demonstrated that women treated with a corset were, on average, 1.4 years older during their initial pregnancy compared with those who underwent surgical interventions [18]. Gestational age at birth was comparable between individuals with AIS and those without AIS. Lebel and Bauchat both documented an average gestational age at birth of 39 weeks, indicating no discernible distinctions between patients undergoing conservative management and those opting for surgical intervention [21,25]. Orvomaa’s cohort of surgically corrected patients revealed that 90% of mothers delivered after 38 weeks, aligning with the general population average [26]. Similar results were observed in Grabala’s investigation [11]. A separate analysis [22] identified minimal discrepancies in the mean count of live births and pregnancies. The average number of live births stood at 2.1 for conservatively managed participants and 2.3 and 2.7 for those managed surgically (fused above L2 and fused below L3, respectively). These findings contrast starkly with the recent research conducted by Ohashi et al. [27], wherein a substantially lower number of offspring was reported among AIS patients (1.3) relative to healthy controls (1.7), even after adjusting for age. Unfortunately, in the available literature, there are no separate publications and no division of nulliparous women into two groups: women who tried to have children but were unable to have children, and women who simply did not try. AIS is known to cause significant mental/emotional impact, and this impact has undoubtedly discouraged at least some women from having children in an attempt (at least in their own minds) to protect their future children from the diseases of scoliosis.

## 3. Back Pain and Pregnancy

Several patients experiencing AIS have documented the emergence of novel back discomfort linked to pregnancy or escalated BP while pregnant; nevertheless, the frequency and intensity of this BP differed across various research studies. Several studies indicated no significant variance in the occurrence of BP among AIS patients and control cohorts [11,18,28,29,30]. Moreover, investigations have shown a resemblance in the prevalence of BP during pregnancy between AIS patients treated surgically and those treated with braces [18], as well as patients who underwent previous spinal fusion procedures compared with those without such interventions. Bivia-Roig et al. [31] highlighted that pregnancy induces modifications in the lumbar motion patterns, resulting in diminished flexion range and reductions in both the proportions of lumbar flexion during spine flexion and the duration that pregnant individuals maintain peak levels of lumbar flexion. These alterations in kinematics are accompanied by variations in the neuromuscular reactions of the lumbar extensor muscles, characterized by heightened activation during spine flexion and a reduction in their myoelectrical silence duration [31]. These observations potentially signify biomechanical adjustments to the heightened anterior load and increased ligament laxity linked to pregnancy, potentially contributing to the onset of pregnancy-related low BP [31]. According to Grabala et al., BP at a comparable level was noted during pregnancy in individuals with a history of conservative treatment, in those with surgical correction for AIS, and in healthy women [11]. The occurrence of BP during pregnancy was found in 48% of the scoliosis and pregnancy group and 34% of the healthy pregnancy control group. Following childbirth, back pain was present in 43% of the scoliosis and pregnancy group and 42% of the healthy control group. Postpartum, the occurrences in these groups were also similar [11]. Nonetheless, the research demonstrated an increased prevalence of low BP in patients who underwent scoliosis surgery and were fused to L3–L4 (up to 40%). In the study by Orvomaa et al. [26], the prevalence of BP in AIS surgery patients was 40%, while in the studies by Danielsson [18,32], it was 36%; in Grabala’s study, this prevalence was 48%. Furthermore, for the group of patients without AIS who delivered babies, the incidence of BP was 34%, whereas in other studies, it was 49% [26]. Recent reports, however, suggest that the prevalence of BP in the general population ranges between 60% and 80%, a figure comparable with that reported in AIS patients [18,26,33]. Patients with scoliosis in the lumbar and thoracolumbar spine exhibit a higher proportion of spine pain (80–86%) compared with patients without scoliosis. In the investigations with a long-term observational period, the prevalence of BP in AIS patients was 77% in contrast to 37% in the control group. Chronic BP was experienced by 61% of the scoliosis patients and 35% of the control group. BP during pregnancy is relatively common in healthy women without a history of surgery or scoliosis, with an estimated impact on around 70% of pregnant women. The severity of the pain can vary, ranging from mild discomfort, hindering the maintenance of a specific body position, to intense pain interfering with independent functionality. The unequivocal cause of the pain cannot be pinpointed, as it arises from various factors like hormonal activity, excessive strain on the spine, alterations in body equilibrium, forward shift in the center of gravity, and accentuation of lordosis [3,18,26,32,34,35,36]. In a relatively limited subset of women (approximately 10%), the pain is a continuation or exacerbation of a pre-existing condition before pregnancy [15,24,31,35]. In rare cases, a new structural spinal ailment may manifest, such as spinal disc herniation. On the whole, BP during pregnancy was frequently reported, albeit for most individuals, the pain was not described as severe or incapacitating. Betz et al. discovered that even though 77% of women with scoliosis experienced BP during pregnancy, only 12% categorized the pain as severe [15]. Falick-Michaeli et al. [24] ascertained that 35% of AIS patients reported severe BP during pregnancy, and among them, 76% highlighted sustained BP affecting their post-delivery life [24]. Similarly, in another study involving patients treated with Harrington instrumentation, approximately 10% necessitated sick leave due to their BP [26].

## 4. Mode of Delivery and Anesthesia

In individuals diagnosed with scoliosis and undergoing treatment utilizing the Harrington technique, the incidence of cesarean deliveries varies from 2% to 52% [24,26,32,35]. Research studies directly comparing the likelihood of CS following segmental spinal instrumentation are currently absent. Among the 18 investigations encompassing patients managed through surgery, or a blend of surgical and conservative approaches, 9 of them documented a higher frequency of epidural and/or spinal analgesia interventions in these patients when contrasted with individuals without health issues. Nevertheless, the successful administration of epidural anesthesia was ultimately accomplished [21,37,38,39,40,41,42,43,44]. As outlined by Grabala and colleagues, 64% of women who underwent instrumented spinal fusion required cesarean delivery [11]. The necessity for CS was more pronounced in females with extended fusion, reaching up to L4. Additionally, these particular patients exhibited a greater demand for general anesthesia during childbirth in comparison with those with shorter fusions. According to the findings of Swany et al. [22], an elongated spinal fusion complicates the management of pain (specifically epidural anesthesia) during the process of childbirth, instigating concerns regarding peripartum pain, consequently heightening the probability of cesarean delivery. Conversely, obstetricians and healthcare practitioners present during deliveries might have been inclined to recommend cesarean section for women with a documented history of spinal fusion due to apprehensions regarding complications during the second stage of labor [11]. Notably, no substantial scientific proof yet exists indicating a notable distinction in CS rates among patients with AIS, irrespective of the mode of treatment—surgical or conservative. Among these studies focusing on surgical cases, elevated rates of cesarean deliveries in patients who underwent surgical instrumentation have been highlighted [11,19,22,26]. While Kino et al. [20] observed two cesarean deliveries in the AIS cohort and none in the healthy control group, the authors mentioned that one CS was decided upon by the physician’s discretion, whereas the other was due to twin–twin transfusion syndrome. Correspondingly, Villevieille et al. [41] reported a cesarean delivery rate of 33% among their sample yet did not draw comparisons with the national average or a control cohort. Three studies omitted any specification of the type of management strategy utilized [25,37,45]. Lebel et al. [25] identified a heightened CS rate in the AIS group compared with controls. On the other hand, Siegler et al. [45] and Smith et al. [37] solely presented the CS rates among AIS participants, which were 17% and 41.4%, respectively. Difficulty in attempting or achieving analgesia was documented in two investigations involving surgical patients [21,39], while one study highlighted an unsuccessful attempt at spinal anesthesia [15]; nevertheless, the treatment history of this particular patient with AIS remained ambiguous. Four studies that included surgical patients documented successful spinal analgesia [46,47,48,49]. Falick-Michaeli et al. [24] revealed that 70% of AIS surgical patients at a singular center study location experienced a refusal of epidural anesthesia by anesthesiologists. This was primarily due to the perceived lack of access to a suitable site for catheter placement. Grabala et al. [11] observed no distinction in the utilization of spinal block for analgesia between the AIS group (surgical) and the control group. The usage of spinal analgesia or anesthetics in conservatively managed AIS patients lacks substantial evidence. Piosik et al. [50] indicated the absence of issues with analgesia or anesthetics in conservatively managed patients. Bauchat et al. compared surgically corrected females with AIS with healthy controls, revealing variations in the type and efficacy of peripartum anesthesia [21]. Traditional epidural anesthesia was administered to 39% of patients with spinal instrumentation compared with 5% of the controls, with the remaining individuals receiving preferred combined spinal anesthesia. Furthermore, instrumented patients required 40% more time to achieve neuraxial anesthesia. Despite these challenges, the rate of failed neuraxial anesthesia in this cohort remained relatively low at 12%, with no severe complications reported similar to other studies [51]. In a cross-sectional analysis by Falick-Michaeli and colleagues, 70% of surgically corrected AIS women faced rejection of spinal anesthesia (attributed to the lack of a catheter access site), in contrast to 0% of the non-AIS controls [24].

## 5. Loss of Deformity Correction and Pregnancy

Assessing the development of curvature after a conservative treatment during pregnancy or the regression of pregnancy-related deformities following scoliosis surgery poses challenges, with varied outcomes based on the assessment and treatment methods employed. Betz et al. approximated that a quarter of patients treated conservatively encounter curvature progression exceeding 5°, with around 10% experiencing over 10° progression [15]. The extent of curvature prior to pregnancy directly influences the progression, with 6% of patients having a less than 30° curvature pre-pregnancy witnessing a 10° or more progression, compared with 29% of those with pre-pregnancy curvatures of 50° or higher. Berman et al. reported that 43% of patients with a 25° curvature observed a slightly detectable progression of at least 5° [52]. Betz et al. highlighted that the patient’s age at first pregnancy does not impact the curvature progression risk [15]. This contrasts Cochran et al.’s findings that patients treated with a corset for AIS and becoming pregnant before 23 years old face increased progression risk [14,53]. Ascani and colleagues established a correlation between the number of pregnancies and the degree of curvature progression, noting average increases of 13–16° after the first pregnancy and 16–23° after multiple pregnancies upon reaching adulthood [17]. In contrast, Blount and Danielsson’s studies indicated no link between the number of pregnancies and curve progression. The data concerning surgically treated patients show slight variations [17,54].

Available studies regarding scoliosis and its impact on pregnancy and childbirth remain limited in the current literature. Accessing these studies has proven challenging due to the diverse range of methodologies utilized, including conservative management or surgical interventions utilizing outdated techniques like the Harrington instrumentation [15,18,26,32,33,34]. Contemporary implant systems designed for correcting spinal deformities, which feature pedicle screws and rods, allow for three-dimensional de-rotation of the spine. Furthermore, when used in conjunction with osteotomy procedures, these systems demonstrate correction rates ranging from 70 to 90 percent in terms of reducing the magnitude of spinal curvature. Numerous factors play a pivotal role in this context, such as the rate of curvature, the density of screws, the surgical technique employed (including osteotomy), the skeletal maturity, manual skills, the surgeon’s expertise, and the patient’s healing capabilities [11,12,15].

Loss of correction deformity (LOC) subsequent to scoliosis surgery using the posterior approach along with spondylodesis has been meticulously examined and elucidated in various scholarly works [6,30,55,56]. Different research studies have reported that at the final follow-up (FFU), the loss of correction (LOC) in the coronal plane ranged from 2.6° to 31° [8,55,57,58]. Factors contributing to the occurrence of LOC, particularly associated with skeletal maturity, have been clearly identified with also implant-related materials [9,10,59]. A study focusing solely on AIS patients, for whom LOC is presumably less impacted by the ‘crankshaft’ phenomenon, conducted by Hwang et al. [8], explored the risk factors correlated with the reappearance of deformity, demonstrating a relatively notable percentage of correction loss (approximately 8%) in skeletally mature individuals like in other studies [8,9,54,55,59,60]. According to Grabala et al., the incidence of LOC was documented in approximately 10% of the total patient cohort. Within this group, the average LOC was around 10° and was predominantly correlated with the curvature magnitude and AVT. Moreover, 14% of patients in both groups exhibited a loss of correction exceeding 10°. These cases predominantly involved female individuals with a Risser stage of 0–2 during the surgical procedure. Conversely, Yamada et al. [54] proposed that when evaluating factors contributing to the loss of correction, particular emphasis should be placed on the accurate fixation level during the planned surgery. A conservative approach was investigated to manage all AIS patients observed before the years 1974 and 1977 [14,18,52,61]. Notably, the type of brace utilized was not specified in the study by Betz et al. [15]. All investigations reported a clinically significant progression of curvature in certain braced AIS patients subsequent to pregnancy [52]. Another study highlighted either the absence of change or a clinically significant advancement in the curvature of four young (≤20 years) braced AIS post-pregnancy patients. Progression was observed to be more probable in patients managed conservatively with a curvature exceeding 25 degrees (Cobb) before pregnancy. Nonetheless, the majority of research findings indicated that, on average, pregnancy did not lead to substantial clinical alterations in curve magnitude among patients who had undergone brace treatment. The additional studies [22,48] delved into the impact of conservative management on curve progression in AIS patients who had experienced one or more pregnancies, albeit without specifying the precise type of conservative approach. In terms of surgical interventions, multiple studies scrutinized the postpartum curve progression in AIS patients who had undergone spinal instrumentation and fusion [13,15,18,46]. Three studies [13,15,46] detailed a posterior surgical strategy, outlining the instrumentation employed: Harrington instrumentation with distraction and Cotrel–Dubousset instrumentation [18,26,46]. A study by Nachemson and Danielsson involved 6–12 months of bracing after surgery [18]. The outcomes in surgically managed patients resembled those in the braced group, with some AIS patients experiencing curve progression following pregnancy. On average, pregnancy did not lead to clinically significant curve progression in patients who had received surgical management.

## 6. Quality of Life Questionnaires

All available findings concerning quality of life after surgical intervention indicated a general sense of contentment, which was consistent among patients with scoliosis and those in the pregnancy group compared with a healthy female control group. Notably, no instances of sexual dysfunction were documented in the research. A particular study spanning 18 years following patients who underwent selective thoracic fusion revealed that the uninstrumented lumbar curve underwent spontaneous correction, with this correction being sustained even 18 years after surgery utilizing selective thoracic fusion (STF) [62]. Although mild degenerative alterations were observable radiographically, the scores related to HRQoL implied that the psychological and functional well-being of AIS patients who underwent STF remained quite favorable in the long run when contrasted with a population matched in terms of age, gender, and BMI. The mean scores for HRQoL, self-perception, and mental health were notably higher in the STF group than the control group. Various factors such as SRS-22r pain and function, Oswestry Disability Index, Numeric Rating Scale, marital status, and number of children showed no significant differences between the groups. However, it is important to note that all disc heights, except for L5-S1, were substantially lower in the STF group [62]. In a different study [63], at the last follow-up of up to 10 years, the STF group exhibited higher overall HRQOL scores compared with the non-selective thoracic fusion (NSTF) group, with statistically significant differences noted between the groups (STF/NSTF) in various aspects such as SRS-30-Mental health, SRS-30-Satisfaction with management, SRS-30-Pain, ODI, SF-12 PCS, VAS back pain, and VAS leg pain. No statistically significant variances were observed in SF12 MCS, SRS-30-Self-image/Appearance, and SRS-30 [63]. In a study encompassing 40 years of follow-up on AIS patients who underwent surgical procedures [22], HRQOL was found to remain stable over the last 13 years among patients with AIS who had undergone spinal fusion(s) between 1968 and 1982. A relatively satisfactory HRQOL was identified within this cohort of patients now in their middle-aged and older years [23]. A comparative analysis between AIS patients who received surgical intervention versus non-operative treatment [39] indicated that those undergoing segmental pedicle screw instrumentation for AIS maintained a high level of HRQoL over a decade-long follow-up. Their HRQoL outcomes were notably superior to those of untreated AIS patients, except in the domain of function. Nonetheless, the HRQoL levels remained below those of healthy controls [64]. In a similar investigation [65] with a 5-year observation period, AIS patients who underwent surgical correction with pedicle screw instrumentation and fusion displayed enhanced back pain and health-related quality of life compared with untreated AIS patients. Patients in the surgical group exhibited comparable health-related quality of life with that of the healthy control group, except for function, which was significantly lower [65]. Yet, another study comparing surgical intervention with hybrid techniques and pedicle screw instrumentation [66] led to the conclusion that pedicle screw instrumentation provided notably superior correction of major curves and postoperative pulmonary function values without neurological complications when juxtaposed with hybrid constructs. Both methods of instrumentation exhibited similar junctional changes and the lowest instrumented vertebra, operative duration, and postoperative SRS-24 outcome scores in the surgical management of AIS [66]. The treated patients, or those who underwent conservative treatment [56], exhibited diminished HRQoL compared with the untreated individuals with idiopathic scoliosis and curves measuring 45° or more. Despite the prevalence of back pain among many patients, the reported disability on the Oswestry Disability Index (ODI) remains constrained. A study by Grabala et al. [6] evaluated the efficacy of surgical interventions for severe scoliosis, highlighting the safety of such procedures. The surgical approach yielded an average correction rate of 59% in deformity, leading to notable enhancements in respiratory function. This was evidenced by improvements in predicted forced expiratory volume in 1 s and forced vital capacity, resulting in clinically and statistically significant advancements in SRS-22r scores, HRQoL outcomes, and reduction in back pain (from 36% to 8%), along with enhanced sexual function. The planned surgical interventions demonstrate the ability to achieve substantial correction of deformities with minimal complications [6]. Furthermore, surgical management significantly elevates the quality of life for patients with severe spinal deformities, enhancing overall functionality across various aspects of life.

## 7. Limitations

This study’s scope is constrained by the various conservative and surgical approaches utilized among patients. Consequently, an effort was made to examine cohorts of scoliosis patients categorized as nulliparous and parous in order to present comparative data. Cesarean section procedures were conducted across a broad spectrum of indications, including cases involving healthy women, with reported frequency rates spanning from 12% to 40% based on diverse references [24,32,35]. The elevated incidence of cesarean sections is linked to the assessment conducted by obstetricians [18,26,33,34,35]. The retrospective nature of this research raises uncertainty regarding whether the heightened cesarean section rates were influenced by the physical attributes of the patients or by specific preferences within the healthcare team. The occurrence of back pain following instrumented spinal fusion during pregnancy poses challenges in terms of precise classification, necessitating extended follow-up periods.

## 8. Conclusions

Pregnant women with a prior history of spinal fusion for AIS can undergo pregnancy and childbirth with no significant escalation in perinatal complications. There is no increase in perinatal complications among AIS patients when juxtaposed with healthy women. Women who have undergone surgical procedures for AIS have been observed to exhibit a prevalence of back pain comparable with that of healthy pregnant women; however, a higher incidence of low back pain is evident when spinal fusion is extended to the L3 or L4 vertebra. Although back pain is a common occurrence in pregnant women with AIS, the spinal alterations induced by pregnancy are typically minor and transient. In comparison with healthy women, individuals with a history of spinal fusion necessitate cesarean sections more frequently in some cases. This likelihood escalates to 55% when the spinal fusion extends to the L4 vertebra. Following the surgical correction of AIS, women display favorable quality of life and sexual function, even after pregnancy. Females who have received pedicle screw instrumentation for adolescent idiopathic scoliosis and have undergone one or multiple pregnancies do not exhibit curvature progression or deterioration during extended follow-up periods in contrast to surgically treated patients who have not been pregnant. The degree of correction loss during pregnancy is lower in previous reports involving pedicle screw instrumentation than in previous reports involving Harrington or hybrid segmental instrumentation. Despite the valuable insights provided by our scoping review, the information regarding the impact of pregnancy on scoliosis is still insufficient.

## Data Availability

The data are contained within this article.
